# Progresses, Challenges, and Prospects of CRISPR/Cas9 Gene-Editing in Glioma Studies

**DOI:** 10.3390/cancers15020396

**Published:** 2023-01-06

**Authors:** Xianhui Kang, Yijian Wang, Pan Liu, Baojun Huang, Baofeng Zhou, Shufang Lu, Wujun Geng, Hongli Tang

**Affiliations:** 1 Department of Pain, The First Affiliated Hospital of Wenzhou Medical University, Wenzhou 325000, China; 2 Department of Anesthesiology, The First Affiliated Hospital, Zhejiang University School of Medicine, Hangzhou 310000, China; 3 Department of Anaesthesiology, The First Affiliated Hospital of Wenzhou Medical University, Wenzhou 325000, China; 4 Wenzhou Key Laboratory of Perioperative Medicine, Wenzhou 325001, China

**Keywords:** CRISPR/Cas9, glioma, immunotherapy, tumor model, mechanism research

## Abstract

**Simple Summary:**

Glioma is the most common primary intracranial tumor.Glioma involves a wide variety of cells and is highly aggressive, so most patients with glioblastoma have a poor prognosis.Glioma involves a wide variety of cells and is highly aggressive, so most patients with glioblastoma have a poor prognosis. CRISPR/Cas9 has the property of precise localization, and more and more studies focus on using it for the treatment of glioma and the exploration of its pathogenesis.This paper mainly discusses the role of CRISPR/Cas9 in the treatment of glioma patients, screening of key targets for clinical prognosis, and exploring the pathogenesis of glioma.The future research prospects of CRISPR/Cas9 in glioma treatment and potential opportunities and challenges are also pointed out. In order to help readers comprehensively understand the application and development of CRISPR/Cas9 in glioma research.

**Abstract:**

Glioma refers to a tumor that is derived from brain glial stem cells or progenitor cells and is the most common primary intracranial tumor. Due to its complex cellular components, as well as the aggressiveness and specificity of the pathogenic site of glioma, most patients with malignant glioma have poor prognoses following surgeries, radiotherapies, and chemotherapies. In recent years, an increasing amount of research has focused on the use of CRISPR/Cas9 gene-editing technology in the treatment of glioma. As an emerging gene-editing technology, CRISPR/Cas9 utilizes the expression of certain functional proteins to repair tissues or treat gene-deficient diseases and could be applied to immunotherapies through the expression of antigens, antibodies, or receptors. In addition, some research also utilized CRISPR/Cas9 to establish tumor models so as to study tumor pathogenesis and screen tumor prognostic targets. This paper mainly discusses the roles of CRISPR/Cas9 in the treatment of glioma patients, the exploration of the pathogenesis of neuroglioma, and the screening targets for clinical prognosis. This paper also raises the future research prospects of CRISPR/Cas9 in glioma, as well as the opportunities and challenges that it will face in clinical treatment in the future.

## 1. Background

### 1.1. Current Glioma-Related Research

The morbidity of malignant central nervous system tumors and other CNS tumors accounted for about 31.5% of all brain tumors from 2000 to 2014 [1], where untimely and ineffective diagnosis and treatment resulted in unbearable sequelae. Central nervous system tumors growing from different sites and tissue types can cause different degrees of sequelae, including various extents and forms of cognition, motor-sensory and intellectual impairment, and sometimes even death [2]. Ostrom QT et al. collated and analyzed data from the CBTRUS database and showed that malignant tumors accounted for 49.1% of central nervous system tumors from 2014 to 2018, with an average of 4.43 deaths per 100,000 people within the four years [3]. The most common central nervous system malignant tumor is glioma, which accounts for 14.3% of all central nervous system tumors and is the main cause of death in most patients with intracranial tumors [4]. As per different cell types, gliomas can be classified into astrocytoma, glioblastoma, oligodendroglioma, and many others, and the treatments and prognosis of different types of gliomas vary accordingly. However, they all are derived from a conventional surgical excision in combination with radiotherapy and chemotherapy [5].

In addition to conventional surgical approaches, scientists are also devoted to seeking new breakthroughs to strengthen the diagnosis and treatment of glioma. Moolten et al. demonstrated that antibody-toxin fusion proteins could target tumor cells [6]. Based on this, Pastan I et al. developed an immunotoxin, that is, a molecule that specifically binds to the receptors that are overexpressed on tumor cells to kill them. The glycoproteins (non-metastatic melanoma protein B) receptor on glioma cells also provided a new idea for the targeted therapy of glioma [7].

At present, the pathogenesis of glioma is not yet clear, and the potential pathogenic factors include heredity or specific gene polymorphism (genetic problem), ionizing radiation, nervous system carcinogens, and others. Therefore, we can consider conducting new research on gene therapies. Currently, the gene therapy field is a hot topic in today’s study on glioma; researchers have discovered through gene sequencing technology that genes, including EGFR and ERT, can be used to diagnose potential glioma patients and determine the prognosis [8]. By gene integration and the analysis of glioma patients, Lake JA et al. found that 39 of 61 cases showed targetable genomic alterations in their genes, 50% of which involved BRAF integration or mutation [9]. Some experiments have also proved that the diagnosis, prognosis, and treatment of glioma can be assisted by identifying mutations in the patient’s genes, such as IDH1 and TP53 [10]. In addition, many researchers have explored the mechanisms and functions of various genes in the pathogenesis and disease progression of glioma [11,12,13,14]. A viral vector developed and based on genetic studies has also been confirmed to have a certain effect on the treatment of patients with recurrent high-grade glioma [15].

As a new gene editing technology, CRISPR/Cas9 can serve as an important tool for gene research in gliomas [16]. CRISPR/Cas9 plays an important role in the immunotherapy of glioma, the establishment of a tumor model, mechanism research, and the screening of targeted drugs for gliomas. Current studies have shown that the CRISPR/Cas9 gene-editing can induce tumor cell autophagy and accelerate its apoptosis and other mechanisms to achieve its purpose of permanently killing tumor cells [17,18], which not only overcomes the medication limits of conventional therapies but also improves the efficacy of the treatment [19,20]. Some researchers also regarded CRISPR/Cas9 gene editing as one of the methods for treating gliomas in animals; they further studied its delivery methods [21,22] and compared its treatment effect with other gene therapy methods that have been discovered [23]. As mentioned above, we believe that CRISPR/Cas9 is expected to be applied in the clinical treatment of glioma and has a bright prospect.

### 1.2. Overview of Studies Related to CRISPR/Cas9 Gene-Editing

With the rapid development of gene editing in recent years, genome editing by manipulating functional DNA sequences in the host genome has become a basic strategy in the field of biomedical research [24]. As a brand new targeted modification technology in genomes, CRISPR/Cas9 (clustered regularly interspaced short palindromic repeats/CRISPR-associated protein 9) has been extensively studied due to its simple system structure and easy operation [25]. The CRISPR/Cas system consists of a series of genes encoding Cas proteins and CRISPR elements, of which the CRISPR elements are composed of multiple identical repeat sequences and different spacer sequences arranged alternately. There are many orthologues in Cas9 proteins, including the CRISPR/SpCas9, which is derived from Streptococcus pyogenes that has been widely studied, the CRISPR/FnCas9 from Francisella novicida and CRISPR/CjCas9 from Campylobacter jejuni [26,27,28]. Due to the certain conservation in genes encoding Cas proteins, researchers have roughly classified the CRISPR/Cas system according to the difference in Cas and identified three main types of the system, that is, Cas3, Cas9, and Cas10, which are effectors, respectively for type I, II, and III systems [29]. Compared with the complex endonucleases in type I and III CRISPRs, the type II CRISPR/Cas system involves the least Cas proteins and is generally considered to be the smallest CRISPR/Cas system, including only CRISPR repeat-spacer sequences and three to four Cas genes. Among them, Cas9 encoded a protein with many domains and of a high molecular weight that could independently target, cleave, and invade DNAs. Over the next ten years, many researchers cast their eyes on the type II CRISPR/Cas system [12,30]. The 0protospacer adjacent motif (PAM) is a short DNA sequence that can be recognized when Cas9 acts on the DNA substrates binding and catalysis required. Otherwise, PAM-presenting oligonucleotides (PAMmers) stimulate the site-specific endonucleolytic cleavage of ssRNA targets, similar to the PAM-mediated stimulation of the Cas9-catalysed DNA cleavage. Using specially designed PAMmers, Cas9 can be specifically directed to bind or cut RNA targets while avoiding corresponding DNA sequences [31].

The earliest study on the CRISPR/Cas9 system can be traced back to 1987, in which Ishino et al. first discovered a cluster of palindromic sequences with short spacers in Escherichia coli [32]. CRISPR/Cas9 initially described as a defense mechanism of bacteria against bacteriophage infection, emerged as a promising tool for genome editing due to the ease of adapting it into mammalian cells and its versatility and flexibility for targeting virtually any genomic loci [33,34,35]. In 2002, such a unique sequence family was officially named CRISPR. Since 2011, the basic functions and mechanisms of the CRISPR systems have become more well-defined, and the systems have subsequently been applied to explore various biotechnologies. In 2013, Hwang et al. designed a sgRNA complementary to the *fh* gene sequence, which achieved the multigene knockout in the embryo of zebrafish with the highest efficiency of 59.4% [36]. In 2014, NIU et al. injected the compounds of CRISPR/*Cas9-sgRNA* into the embryos of a cynomolgus monkey using the single-cell embryo microinjection technology to edit Ppar-g and Rag1, effectively achieving gene modification on a specific site [37] (Shown in Table 1). The universality and high efficiency of genome editing in various cells and organisms with the CRISPR/CAS9 system prove the huge advantage of the system in genetic modification, making it an efficient, convenient, and accurate gene-editing tool for a new generation.

**Figure 1:**
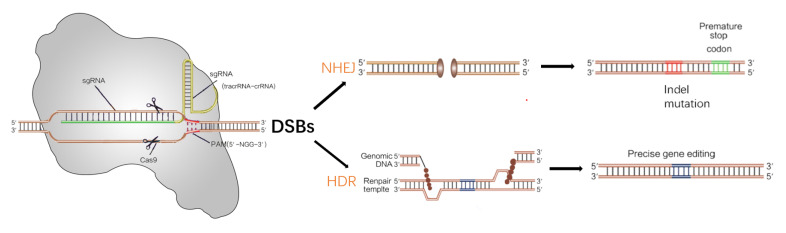
Gene editing mechanisms of the CRISPR/Cas9 system. DSBs (DNA double-stranded breaks) induced by Cas9 (brown) can be repaired in one of two ways: the non-homologous end joining (NHEJ) pathway or the homology-directed repair (HDR) pathway.

CRISPR interferes and defends against foreign organisms mainly through three steps. Firstly, foreign genes are identified. After the foreign DNA is identified by the host cells, a short fragment (30–50 base pairs) is inserted into the CRISPR site of the host cells as a spacer sequence. Then, crRNA is formed during transcription after the nucleotides have processed what was originally transcribed (pre-crRNA), it enters a RNAs (crRNAs) bank derived from short CRISPRs, and each crRNA contains a sequence complementary to a foreign DNA. The two RNAs, that is, crRNA and tracrRNAtrans-activating crRNA, are integrated into a sgRNA. Finally, the Cas9 protein combines with sgRNA to form a ribonucleoprotein complex, which recognizes and cleaves target gene sites [41,42,43,44,45]. After the Cas9-sgRNA complex identifies the target gene site, the target double-stranded DNA unwinds to make sgRNA bind with the target DNA sequence in the seed region, thereby forming an R-loop structure, which activates the cleavage site for cutting the target strand from the non-target strand in the double-stranded DNA to produce double-strand break (DSB) DNA fragments. DSB is achieved by non-homologous end joining (NHEJ) to generate a random insert mutation or deletion mutation or is repaired by homology-directed repair (HDR) in the presence of foreign DNA fragments so as to accurately integrate the target sequence into the genome of the target cells (Figure 1) [39,46]. NHEJ can introduce the deletions or insertions of multiple bases, resulting in damage to the sequences of the transcriptional activation-related promoter, enhancers, and open reading frame, thereby achieving the knock-out of a specific gene. HDR could cause the mutation of a specific gene or a predictable knock-in in the presence of a foreign DNA template [47].

## 2. Roles of CRISPR/Cas9 in Glioma

**Figure 2:**
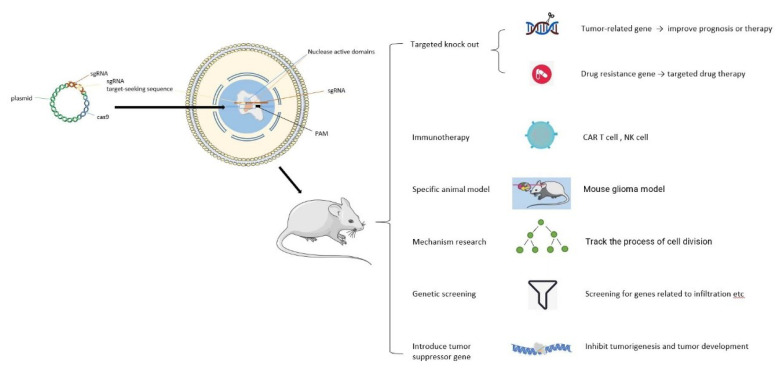
Advances in the research on and application of CRISPR/Cas9 in Glioma. CRISPR/CAS9 was introduced into a mouse model bearing glioma to impact the formation, progression, and treatment of glioma by different modes of action.

CRISPR/Cas9 is a two-component system that includes Cas9 and sgRNA, which play roles in the protein and RNA, respectively. Clusters of regularly interspaced short palindromic repeats (CRISPR/Cas9) have been widely used in various fields of biology and medicine [48,49]. With the development of gene sequencing and editing, gene sequencing technology has become increasingly and widely applied in basic research, and the basic research on CRISPR/Cas9 in glioma (Table 2) is shown in Figure 2.

### 2.1. Inhibition of Glioma Progression by Enhancing Expression of Tumor Suppressor Genes

CRISPR/Cas9 gene-editing can not only introduce tumor suppressor genes into tumor cells but also target the repair of mutated tumor suppressor genes and ultimately restore and improve the activity and function of tumor suppressor genes, thereby inhibiting the occurrence and progression of tumors. The TP53 gene encodes the transcription factor and oncosuppressor p53 protein that regulates a multitude of intracellular metabolic pathways involved in DNA damage repair, cell cycle arrest, apoptosis, and senescence. A majority of TP53 mutations are missense, which brings immense opportunities for the CRISPR/Cas9 system, and have been successfully used for correcting single nucleotides in various models both in vitro and in vivo [64]. In addition, using the CRISPR-Cas9 system can improve the efficiency of accurate gene correction and insertion [50].

### 2.2. Targeted Gene Knockout for Treatment of Glioma

The mutation of the gene was closely related to the occurrence and prognosis of glioma [51]. At present, research is focusing on the use of CRISPR/Cas9 in the treatment of glioma by knocking out tumor-related genes. The chromatin assembly factor 1 subunit A (CHAF1A) is highly expressed in glioblastoma cells, and the highly expressed CHAF1A is related to poor prognosis following glioblastoma treatment, which further promotes the proliferation of glioblastoma cells. Honghai Peng et al. used CRISPR/Cas9 to knock out CHAF1A, resulting in G1 phase arrest and the apoptosis of glioma cells (U251 and U87). The inhibition of CHAF1A also blocked the AKT /FOXO3a /Bim signaling pathway for glioblastoma cell proliferation. This finding suggested that CHAF1A might be a new therapeutic target for improving the prognosis of glioblastoma [50] and could possibly develop into a target of glioblastoma drugs and a prognostic biomarker. Li-Na Zhou et al. knocked out Ninjurin2 shRNA (via CRISPR/Cas9 gene-editing) in established primary human glioma cells, which effectively inhibited cell survival, growth, proliferation, migration, and invasion while inducing the activation of apoptosis [65]. The IGF2BP1 knock-out induced by CRISPR/Cas9 also activated MAGEA6-AMPK signaling, leading to the apoptosis of glioma cells (A172) [52].

Natural killer (NK) cells are effective cytotoxic effector cells that can fight against glioblastoma (GBM) cells induced by tumor cells. The knock-out of TIM3 with CRISPR/Cas9 enhanced the cytotoxicity of human NK cells to GBM cells. The knock-out of TIM3 mediated with CRISPR/Cas9 could also be a promising immunotherapy method for the treatment of GBM [53]. Vacuole membrane protein 1 (VMP1) is an important autophagy-related protein that plays a role in tumor genesis and progression. CRISPR/Cas9 gene-editing resulted in the deletion of *VMP1*, which significantly inhibited cell proliferation, increased cell apoptosis, and induced cell cycle arrest. The knock-out of *VMP1* blocked the autophagic flux, thereby making glioma cells sensitive to radiotherapy and chemotherapy [54].

### 2.3. Research on Targeted Drugs for Glioma

Targeted oncotherapy is our common goal as targeted therapies can more precisely target the tumor and avoid damage to normal tissues as much as possible, thereby increasing the prognosis and quality of life of the patients [55,66,67,68]. Xiangpan Li et al. [40] proposed computational tools based on CRISPR/Cas9 to predict the clinical outcomes of a patient with low-grade glioma. They suggested that CRISPR/Cas9 could not only be used to treat patients but also select patients who could have better clinical outcomes. In recent years, CRISPR-based targeted therapy tools have developed rapidly, including gene activation (CRISPRa) or inhibition (CRISPRi) based on CRISPR. CRISPRa is very helpful in screening gain-of-function, while CRISPRi is more powerful in screening function loss than the RNA interference (RNAi) bank [23,69]. Positive selection using the CRISPR library can detect viable cells with specific conditions, such as pharmaceutical research on tumor cells, and can easily elucidate the mechanism of drug resistance [70]. On the other hand, negative selection can effectively detect dead cells or slow-growing cells and can identify the genes necessary for survival, which may be a promising candidate for molecularly targeted anti-tumor drugs. In addition, negative selection can also be applied to compound lethal interactions, in which the perturbation of both genes results in a loss of viability that is not influenced by any single gene. This mechanism is very important for determining the optimal portfolio of molecular targeted therapies for tumor drugs [71]. Since TMZ is the first-line chemotherapy drug, and the mutation of ATRX resulted in the most commonly abnormal heredity in gliomas, Han Bo et al. knocked out ATRX to explore the influence of ARTX on TMZ resistance [72].

### 2.4. Application in Immunotherapy of Gliomas

**Figure 3:**
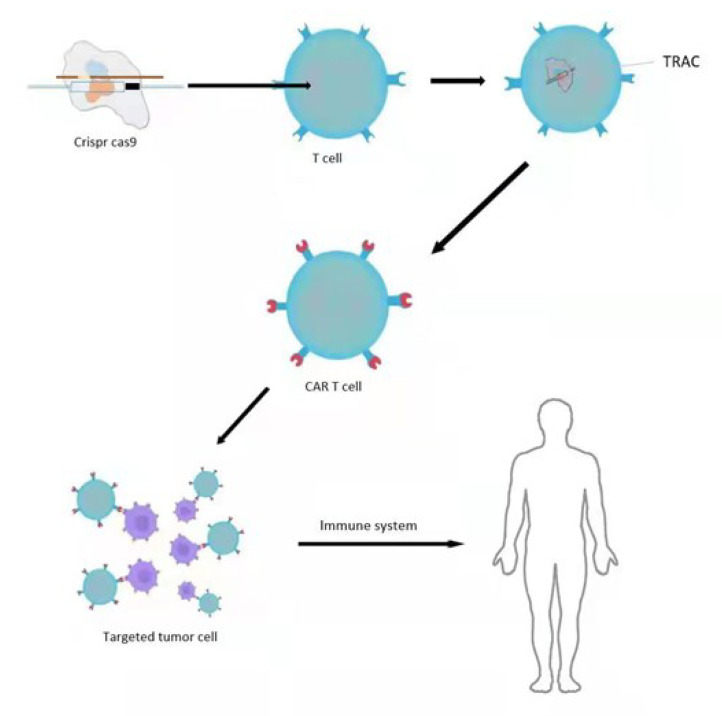
Production of CAR-T Cells. CRISPR/CAS9 was applied in the CD19-specific CAR targeted T-cell receptor α constant (TRAC) loci to result in the uniform CAR expression in human peripheral blood T lymphocytes, and later, the CAR-T cells selectively attacked tumor cells to achieve the treatment effect.

Tumor genesis is driven by a variety of genetic alterations and is always related to complicated immune responses involving the immune evasion of tumor cells and the production of an immunosuppressive tumor microenvironment [73]. Tumor immunotherapy is composed of antibodies, adoptive T-cell infusion, vaccines, and cytokines, which is a new strategy to combat cancers by artificially stimulating the immune systems. It has developed rapidly in recent years, has significant curative effects in hematopoietic malignancies and solid tumors, and is regarded as one of the most promising cancer treatments [74]. However, currently available immunotherapeutic agents have high toxicities and low success rates, and, with the continuous emergence and development of tumor cells and tumor suppressor genes, the immunotherapy that is suitable for multi-target therapies is only limited to a few carcinogenic pathways [75,76]. As a specific gene editing tool, CRISPR/CAS9 is applicable to correct pathogenic mutations with minimum toxicity and can also be used as an adjuvant for immunotherapies to achieve a more robust immune response [77]. In addition, one of the most promising applications of CRISPR/CAS9-mediated genome editing in tumor immunotherapies is the production of CAR-T cells [78,79]. CAR usually contains a variable fragment of the extracellular single strand for identifying and binding a specific tumor-associated antigen and an intracellular chimeric signaling domain for driving the activation of T cells [80]. Using CRISPR/CAS9 on the CD19-specific CAR-targeted T-cell receptor α constant (TRAC) site not only results in uniform CAR expression in human peripheral blood T lymphocytes but also enhances the activities of T cells, which are much better than those CAR-T cells conventionally produce in acute lymphoblastic leukemia (ALL) mouse models [56,81] (Figure 3).

Some studies also utilized CRISPR/Cas9 to disrupt signaling via PD-1 in primary human T cells and to create potentially “ready-made” allogeneic CAR-T cell products by simultaneously editing the site of TRAC and B2M [75,76,77]. The immune checkpoint mediated by PD-1/PD-L1 is one promising therapeutic target in GBM [82]. Some studies have shown that PD-1 signaling is down-regulated by CRISPR/Cas9 in mice born with glioblastoma, which improves the efficacy of CAR-T therapy [83].

Homolog 6 of B7 (B7-H6) is a newly discovered member of the B7 co-stimulatory molecule family. When binding with NKP30 of NK cells, B7-H6 can mediate the regulation of immune responses by NK cells. Some research also studied immunotherapy targeting glioma stem cells by knock-out B7-H6 with CRISPR/Cas9 gene-editing [57].

### 2.5. Assistance on the Establishment of Specific Animal Models

A large number of studies have discovered the complexity of the genome genetics of cancer patients. Only a few genes are mutated at a high frequency (>20%), but most cancer genes in most patients have a mutation frequency of between 2% and 20% or less [84,85]. As a focus of tumor research is to functionally validate the candidate gene changes related to cancer progression and treatment response [58], it is necessary to develop a more flexible model, that is, a complex animal model containing a variety of genetic changes for use in preclinical trials to expedite the identification of tumor driver genes.

The continuous improvement of genome engineering technology has made the in vivo manipulation of all candidate genes possible. Combining the RCAS-TVA system and CRISPR/Cas9 gene editing tool can precisely establish human tumor models [59,86]^,^ which can be established with CRISPR/Cas 9 by the inactivation of a tumor suppressor gene [87,88], somatic point mutation [89,90], and complicated genome rearrangement [84,85]. Carlson Jeff C et al. established a natural mouse glioma model with immune activities utilizing in-utero electroporation and the CRISPR/Cas9 technique to study the three-dimensional dynamics of vascular tumors during glioma progression [58].

### 2.6. Screening of Specific Functional Genes

Glioblastoma is highly malignant among neurogliomas, with high tissue invasion and high mortality. In order to better understand the invasion processes of glioblastoma, Prolo Laura M et al. first introduced the large-scale use of CRISPR/Cas9 for screening of loss of function, which aimed at screening genes that could promote tumor cells invading normal tissues, and they found MAP4K4 to be a new potential target for constraining the invasion of glioblastoma [52].

Since it is difficult to understand the mechanisms of growth and survival of tumorigenic population glioblastoma, targeted treatment is impossible. Therefore, some studies have utilized CRISPR/Cas9 in GBM stem cells (GSC) from patients to explore the functions of encoding genomes and determine the pathways related to the growth of tumorigenic populations to reveal the pathways necessary for genes related to the proliferation of GMB and to better understand the growth and drug-resistance of GBM [59].

Furthermore, glioblastoma is highly malignant, and at present, only a few clinical approaches can effectively treat glioblastoma. Temozolomide (TMZ) is one of a few chemotherapy drugs that can treat glioblastoma, which has very limited effects in later stages due to the resistance of tumor cells [86,91]. Rocha Clarissa Ribeiro Reily et al. introduced the CRISPR/Cas9 whole-genome lentivirus screening library into the human glioblastoma cell line to knock out or activate genes in order to identify the genes regulating the resistance of glioma cells to TMZ. Then, a few candidate genes were screened by screening the gene knock-out or activation that can be targeted by inhibitors or small molecules to weaken the resistance of glioblastoma to TMZ, some of which have been clinically used [60].

### 2.7. Mechanism Research on Glioma

Glioma is one of the most aggressive human malignancies, with both high morbidity and mortality. In clinical practice, it is usually diagnosed as advanced with a very poor prognosis [92,93,94]. Studies on the mechanisms of genesis and progression of glioma are very important for improving the prognosis of glioma patients [95,96,97]. Ogawa Junko et al. used CRISPR/Cas9 technology to target an HRas-IRES-tdTomato construct by homologous recombination into the TP53 locus to observe the glioma initiation in human organoids [61]. The aryl hydrocarbon receptor (AHR) plays an important role in maintaining cellular environmental homeostasis and pathophysiology, and some research knocked out *AHR* in glioma with CRISPR/Cas9, which enhanced the invasiveness of glioma cells in the mouse xenograft model and up-regulated the expressions of genes that correspondingly promoted invasion or migration [98]. DAZL is a cancer germline gene, which can promote tumor transformation, and plays important roles in cancer genesis and progression. The knock-out of DAZL in the glioblastoma cell line with CRISPR/Cas9 gene-editing technology found that DAZL contributed to the tumorigenicity of glioblastoma by reducing cell stemness [14]. The potential pathogenesis of glioma can also be studied with CRISPR/Cas9 gene-editing technology by mediating the directed mutation of glioma cells. Some studies utilized CRISPR/Cas9 in combination with piggyBac transposase lineage labeling to module glioma resulting from the damage to neurodevelopment and somatic mutation of neural progenitors [99]. Tejero Rut et al. engineered patient-derived GBM cells by the CRISPR/Cas9-assisted knock-in of an inducible histone2B-GFP (iH2B-GFP) reporter to track cell division history and further study the mechanism of GBM recurrence [63].

## 3. Challenges

Although the CRISPR/Cas9 has been widely used, it still faces some shortcomings and needs to be improved.

(1) CRISPR/Cas9 has off-target effects during operation.

CRISPR/Cas9 has an off-target effect during operation because the specificity of DNA and RNA is not absolute. Similar to the siRNA, the specificity of siRNA is not absolute, and off-target gene silencing can occur through different mechanisms. Many methods have been published to solve this problem; it is usually thought that this problem can be mitigated by achieving consistent results through the targeting of different sequences in specific genes with several different siRNA. For some biological problems, the CRISPR-Cas9 system may be superior to other solutions. This is because shRNA or siRNA can affect cell function, while CRISPR-Cas9 does not. With respect to the off-target effect in gene expression [100], combining CRISPR-Cas9 with shRNA (or siRNA) can distinguish off-target effects from potential compensation mechanisms [101]. Moreover, genetically engineered high-fidelity Cas9, hyper-accurate Cas9 (HypaCas9), as well as enhanced specificity Cas9 (eSpCas9) can reduce off-target activity [102,103].

(2) CRISPR/Cas9 is not precise enough, and genome editing tool-induced DNA double-strand breaks (DSBS) can be repaired by a non-homologous terminal junction (NHEJ) and homologous recombination (HR), but the deflection of the former path in humans and other mammals often leads to imprecise repair [104]. Cas9 sometimes cuts DNA sequences that are similar to the ones it is looking for, but those sequences contain multiple different bases, which can lead to new mutations.
(3) Efficiency and safety of the CRISPR/Cas9 system in the human body. If unedited tumor cells grow faster, the benefit of gene-editing therapy will be diminished.
(4) The tumor is heterogeneous.

Intracranial metabolism is complex and flexible, and brain tumors are affected by metabolism [105]. The types of gliomas are very large and varied [106], and for different patients with the same tumor, as well as the differences between primary and recurrent tumors at different stages of tumor development in the same patient [107], their gene mutations are different, which makes the use of CRISPR/Cas9 for targeted gene therapies face greater challenges.

## 4. Conclusions and Outlook

The conventional approach for glioma focuses on surgical excision in combination with radiotherapy and chemotherapy. Although such an approach can well excise tumor tissues, it also leads to much damage in brain tissues with lesions that are subject to relapse.

Since the occurrence and progression of glioma are closely related to gene polymorphism, the research on gene-targeted therapy has made great progress. Tumor-targeted therapy is an approach for the treatment of tumor gene mutations at a molecular level, and tumor-targeted therapy is achieved by blocking cell signaling pathways related to tumors. CRISPR/Cas9 is a gene therapy that can treat many kinds of diseases by DNA splicing technology, which is currently used in oncotherapy studies. For example, MAGEA6-AMPK signaling is activated by knock-out IGF2BP1 with CRISPR/Cas9, resulting in the apoptosis of glioma cells (A172) and inhibiting the survival of human glioma cells, so as to achieve a therapeutic effect [108].

In this paper, we mainly discuss the use of CRISPR/Cas9 in glioma for immunotherapy, glioma model construction, mechanism research, and the exploration of targeted drugs. The CRISPR/Cas9 platform is so wonderful that it may be an unprecedented technological leap in genome editing for both biomedical research and therapeutic discovery. However, CRISPR/Cas-mediated gene editing is related to chimeric antigen receptor T cells (CAR-T) therapies because most of them are generated using ex vivo editing approaches. Fortunately, scientists such as Lau CH [109] and Pan YC [110] apply themselves to develop delivery strategies that have high tissue specificity, high editing efficiency, and, most importantly, minimized toxicity. At present, most of the gene therapy for glioma is still resting on animal experiments and has not been widely used in clinical practice. However, since 2015, more than 30 clinical trials have been conducted using CRISPR/Cas9 genome editing technology, and the results indicate that patients may achieve positive results [38]. It is believed that in the future, research on the use of CRISPR technology to target the treatment of glioma will continue to develop and will have a wide range of application prospects.

## Figures and Tables

**Table 1:** Discovery history of CRISPR/CAS9 system.

Year	Related Researches on CRISPR/CAS9
In 1987	Ishino et al. first found a clustered palindromic sequence with short spacers in Escherichia coli [32].
In 2002	Such a sequence family was officially named CRISPR by Jansen et al.
After 2011	The mechanism of CRISPR-Cas in the bacteria-acquired immune system was basically elucidated, thus laying a solid foundation for further application.
In 2013	Hwang et al. realized multigene knock-out in the embryo of zebrafish with efficiency at the highest of 59.4% [36].
In 2014	NIU et al. edited Ppar g and Rag1 genes, effectively realizing gene modification on specific loci [37].
Since 2015	CRISPR/CAS9 is beginning to be used in clinical studies [38].
In 2017	The smallest CRISPR/CAS9 was found in Campylobacter jejuni [28].
In 2018	CRISPR/CAS9 is beginning to be used for the study of autophagy [18].
In 2020	CRISPR-Cas9 using targeted lipid nanoparticles is being used for cancer therapy [22].
In 2021	Harnessing the type I CRISPR-Cas systems for genome editing in prokaryotes [39].
In 2022	Based on CRISPR/Cas9 to predict the clinical outcomes of patients with low-grade Glioma [40].

**Table 2:** Research on application of CRISPR/Cas9 in Neuroglioma.

Research Direction	Locus of Action by CRISPR/Cas9	Effect	References
Inhibition of tumor progression by enhancing the expression of cancer suppressor genes	RNA inhibition in combination with CRISPR/Cas9	Could be used for identifying possible off-targets and taking a potential compensating action	[50]
Targeting gene knockout	Knock out CHAF1A	Result in G1 phase arrest and apoptosis of glioma cells (U251 and U87) as well as blocking the AKT/FOXO3a /Bim signaling pathway	[51]
	Knock out Ninjurin2 shRNA	Inhibit the cell survival, growth, proliferation, migration, and invasion while inducing apoptosis	[52]
	Knock out IGF2BP1	Activate the MAGEA6-AMPK signaling, resulting in the apoptosis of glioma cells (A172)	[53]
	Knock out TIM3 gene	Enhance the cytotoxicity to GBM cells medicated by human NK cells	[54]
	Knock out VMP1	Block the autophagic flux so as to make GBM cells sensitive to radiotherapy and chemotherapy	[55]
Research on drugs targeting neuroglioma	Knock out ATRX gene	Explore its influence on TMZ resistance	[53]
Immunotherapy	CD19-specific CAR targeting TRAC loci	Not only result in uniform CAR expression in human peripheral blood T lymphocytes but also enhance the activities of T cells	[56]
	Down-regulate PD-1 signaling	Enhance the efficacy of CAR- T immunotherapy	[13]
	Knock out B7-H6	Study the immunotherapy targeting of glioma stem cells	[57]
Establishment of specific animal models	Specific disease genes	Establish a natural mouse glioma model with immune activities	[58]
Screening of specific functional genes	Screen genes that could promote tumor cells to invade into normal tissues	Explore a therapy to block tumor invasion	[53]
	Explore functions of encoding genomes	Determine pathways related to the growth of a tumorigenic population	[59]
	Screen a few drug-resistance-related genes of which candidate genes can be targeted by inhibitors or small molecules	Weaken resistance of glioblastoma to TMZ	[60]
Research on tumor mechanism	Target an HRas-IRES-tdTomato construct by homologous recombination into the TP53 gene locus	Observe the tumor progression in human organoids	[61]
	Mediate the directed mutation of neuroglioma cells	Study the potential pathogenesis of neuroglioma	[62]
	Knock in an inducible histone 2B-GFP (iH2B-GFP)	Track cell division history and study pathogenesis	[63]
	Knock out DAZL gene	Find out that DAZL contributes to the tumorigenicity of glioblastoma via reducing cell stemness	[14]

## Abbreviations

The following abbreviations are used in this manuscript:

CRISPR/Cas9	clustered regularly interspaced short palindromic repeats/CRISPR-associated protein 9
DSB	double strand break
NHEJ	non-homologous end joining
HDR	homology directed repair
CHAF1A	chromatin assembly factor 1 subunit A
NK	Natural killer
GBM	glioblastoma
VMP1	Vacuole membrane protein 1
TRAC	T-cell receptor α constant
B7-H6	Homologue 6 of B7
GSC	GBM stem cells
TMZ	Temozolomide
AHR	aryl hydrocarbon receptor
iH2B-GFP	inducible histone2B-GFP
